# Construction of Innovation Behavior of College-Student Entrepreneurs Using Entrepreneurship and Innovation Theory Under Educational Psychology

**DOI:** 10.3389/fpsyg.2021.697924

**Published:** 2021-08-30

**Authors:** Baoge Zhang, Song Han, Qiuyan Xu, Lan Jiao

**Affiliations:** ^1^Faculty of Teacher Education, Ningbo University, Ningbo, China; ^2^Faculty of Education, Northeast Normal University, Changchun, China; ^3^Department and Institute of Psychology, Ningbo University, Ningbo, China

**Keywords:** entrepreneurship and innovation education, entrepreneurship, innovation behavior, venture performance, innovation attitude and innovation performance

## Abstract

The purpose is to analyze the entrepreneurship and innovation education of colleges from the perspective of educational psychology and optimize the teaching mode reform of entrepreneurship and innovation courses. In this study, the theoretical research and case studies are combined to explore the performance of college-student entrepreneurs during college and work and have provided data for targeted entrepreneurship and innovation education in the schools. Meanwhile, the specific manifestation of the entrepreneurial spirit during work is analyzed, and the impact of entrepreneurial spirit is discussed on the new venture performance. The case study shows that most of the surveyed college-student entrepreneurs have a higher educational background and short venture-creation time, and their ventures are mostly small and medium-sized enterprises (SMEs) with rapid development. Most entrepreneurs show a strong entrepreneurial spirit during college. Among them, the average score of honesty is 3.85. At work, the surveyed entrepreneurs have high innovation attitude and innovation intention. Most entrepreneurs use innovative methods to solve practical problems in their work, and innovation spirit plays an important role in improving venture performance. Innovation attitude and innovation performance have a significant positive impact on innovation behavior. The research is comprehensive, and the results have very important application value. The results can provide scientific and effective references for colleges to reform entrepreneurship and innovation education.

## Introduction

At this stage, China vigorously promotes mass entrepreneurship and innovation (hereinafter referred to as entrepreneurship and innovation) policy and especially focuses on the entrepreneurship and innovation education of college students (Gideon et al., 2017). The content related to entrepreneurship and innovation policy is written into the government work report to further improve the attention to the innovation and entrepreneurship and innovation education of college students, such as giving full play to the advantages of rich human resources, adhering to the reform and innovation, vigorously cultivating the professional spirit, and cultivating and expanding the new momentum. With the continuous advancement of policies, the number of self-employment in China has increased significantly (Lee and Nathan, 2017). College students, rich in knowledge, vitality, and enthusiasm, have become an important part of entrepreneurial groups under the influence of the entrepreneurial boom. The number of college entrepreneurs has increased year by year and has gradually occupied the majority of entrepreneurs (Antal et al., 2017). In this context, colleges have also vigorously expanded entrepreneurship and innovation education and strive to further improve the awareness among college students for entrepreneurship and innovation and entrepreneurial competency through related courses and competitions, such as the entrepreneurship design competition, entrepreneurship theoretical foundation training, and entrepreneurship lectures. However, some colleges lack the necessary attention in the ideological education of entrepreneurship and innovation. The ideological education of entrepreneurship can help students to thoroughly understand the nature of entrepreneurial activities and cultivate entrepreneurship of college students, so its importance in entrepreneurship and innovation education cannot be overemphasized (Edwards-Schachter et al., 2015). More importantly, contemporary college students have a strong self-awareness. The psychological status of the college students should be fully considered in the development of entrepreneurship and innovation education, and entrepreneurial education courses should be formulated based on scientific research theories. Therefore, educational psychology should be incorporated into entrepreneurship and innovation education, together with other professional disciplines, for targeted entrepreneurship and innovation curriculum reform. This research direction is also a hot spot in related fields (Wu and Song, 2019).

Entrepreneurship and innovation education is also essential for entrepreneurs at work. Currently, with a faster pace of life and work, the business environment is also changing rapidly. To win the upper hand in the fierce market competition, enterprises must master the core competitiveness (Sieja and Wach, 2019). Innovation is an important component of the core competitiveness of enterprises and can determine the development of enterprises. The main work of the entrepreneurs include setting business objectives, arranging management regulations, formulating strategic decisions, shaping and disseminating corporate culture, and exploring the direction of enterprise innovation and development (Lee and Lee, 2016). Entrepreneurs are the pioneers of an enterprise, and they affect the enterprise from all aspects: they can either innovate by themselves or provide key support for the innovation activities to enhance enterprise innovation. Research shows that the innovation ability and awareness of entrepreneurs are key to enterprise innovation (Ng et al., 2016). Therefore, the exploration of innovation ability and awareness of entrepreneurs can promote the healthy development of new ventures, the growth of the industrial economy, and the optimization of social resources as a whole.

Hence, college-student entrepreneurs are studied, and their entrepreneurial spirit and creativity during school and work are explored through questionnaire surveys (QS) and case analyses. Then, corresponding scales are designed with scores and related characteristics for analysis (Deng et al., 2021). In this study, the purpose is to provide an effective reference for targeted entrepreneurship and innovation education in colleges and to explore the impact of entrepreneurial spirit on the innovation and development of new ventures. In the study, the school days capability and working days ability of entrepreneurs are innovatively compared and analyzed. Specifically, the mechanism of entrepreneurial spirit is analyzed throughout the process, which has significant practical application value.

## Materials and Methods

### The Basic Meaning of Entrepreneurship and Innovation Theory

The entrepreneurship and innovation theory is widely used in the research of pedagogy (Colombo et al., 2016). Entrepreneurship and innovation education is the practice to create new things. It can either refer to the education to create new occupations and job opportunities or can refer to specific works in course system, work-integrated learning, school-enterprise cooperation teaching evaluation system, and school status management system (Huggins and Thompson, 2015). Entrepreneurship and innovation education focuses on actively adapting to economic development, unlike the traditional education model. In entrepreneurship and innovation education, students are not merely taught with the theories of entrepreneurship and innovation but are provided with scientific guidance for the innovative ideas and entrepreneurial ability that include many aspects as shown below (Lortie and Castogiovanni, 2015). Entrepreneurship and innovation education can deliver systematic knowledge that helps learners avoid specific risks and help them make scientific planning (Wei et al., 2019). Finally, entrepreneurship and innovation should be fully practiced, which is a major way for effective entrepreneurship and innovation education. Various entrepreneurial activities should be promoted to combine theory with practice and apply knowledge in practical skills (Chen, 2019).

### Analysis of the Entrepreneurship and Innovation Courses in Colleges Based on Educational Psychology

Educational psychology plays a very important role in college education courses. Educational psychology can promote course objectives and the all-around development of the students through a timely understanding of psychological status and clear phase-training goals of the students. From the perspective of educational psychology in colleges, administrators, teachers, and students at all levels should work together to promote the development of entrepreneurship and innovation education (Robinson and Marino, 2015). The school administrators need to unify their understanding and firmly believe that the development of entrepreneurship and innovation education can improve the quality of personnel training. Only through reform on education and learning concepts, entrepreneurship and innovation education can get fruitful results. From the perspective of college teachers, the understanding and recognition of entrepreneurship and innovation education of teachers can determine their teaching behavior. College teachers should be trained more professionally, and they should guide students to participate in the related competitions (Zhao et al., 2018) so that entrepreneurship and innovation education can be improved. From the perspective of students, they are the subject of education, so the ultimate goal of entrepreneurship and innovation education is to help college students master the comprehensive quality of entrepreneurial spirit (Tan, 2020). College students should cultivate their interests and hobbies, strive for opportunities for learning and practice, and cultivate their ability to innovate so that they master the entrepreneurial spirit. Only in this way, can the creative enthusiasm of college students be further aroused (González-Pernía and Peña, 2015).

**Figure 1:**
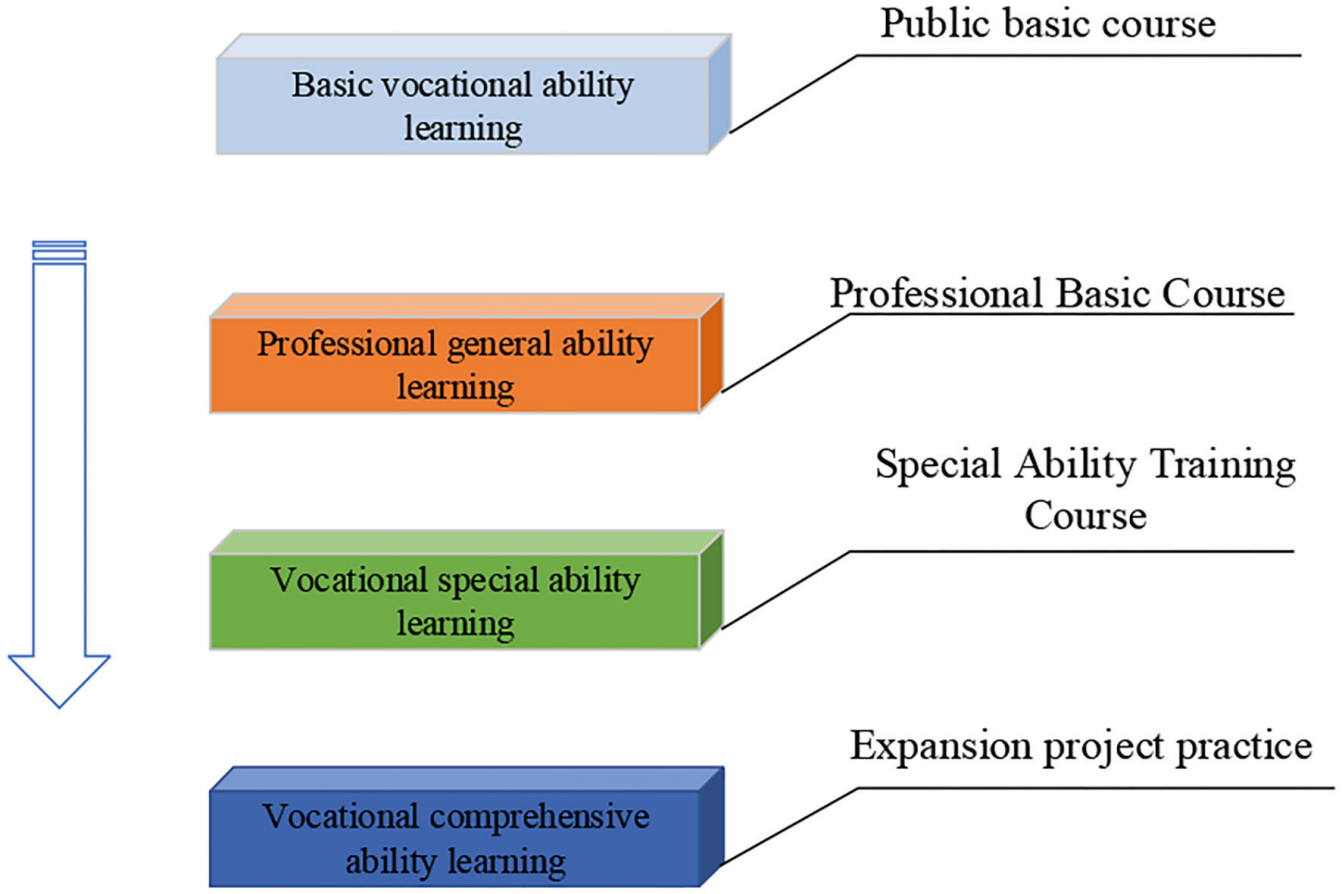
College entrepreneurship and innovation course based on hierarchical progression.

The establishment of practical courses in colleges has always been the focus of colleges, and the hierarchical and progressive practical teaching course system can build up a set of entrepreneurship and innovation courses in colleges (Bunten et al., 2015). This learning method focuses on research learning and autonomous learning, which fully mobilizes the learning enthusiasm of the students. The proposed teaching system has effective applicability on entrepreneurship and innovation courses that require active learning of the students. The structure of the system is shown in Figure 1.

Colleges should reasonably allocate resources and supervise the teaching process seriously during entrepreneurship and innovation education. The teaching process should be student-centered and practice-oriented (Pisoni, 2019). Entrepreneurship and innovation education aims to cultivate awareness of entrepreneurship and innovation in the students and comprehensively improve the quality and related abilities in them. Besides, entrepreneurship and innovation education should be market-oriented to combine specialties and positions. Under a constantly mutating market economy, students should be cultivated with ideas and skills to keep pace with the times to adapt to the development of modern society (Jodoin, 2017).

### The Features and Content of College-Student Entrepreneurial Spirit

Especially, in the context of poor employment rates, entrepreneurship and innovation education can alleviate the employment pressure of college students, promote social stability, unity of people, and economic development (Urbano et al., 2019). Some entrepreneurs even have improved their entrepreneurial ability through the entrepreneurship and innovation course. Colleges should pay more attention to entrepreneurship and innovation education for talent cultivation (Doern et al., 2019). The entrepreneurship and innovation courses in colleges should be up to date. With an ever-faster information surge and knowledge expansion, the multidisciplinary exchange has also become part of entrepreneurship and innovation education (Fu et al., 2019). Thus, supplementary courses are added to entrepreneurship and innovation education as well. Meanwhile, the teachers with practical experiences have been entrusted in entrepreneurship and innovation education to provide good guidance for students (Feng et al., 2020).

The entrepreneurial spirit is the core content of entrepreneurship and innovation education (Sutter et al., 2019). The definition of entrepreneurial spirit varies, and some regard it as the innovative spirit that lays the spiritual foundation of entrepreneurs (He et al., 2019). College students should be cultivated with an entrepreneurial spirit. In addition to the innovation spirit, the entrepreneurial spirit also contains dimensions, such as leadership, collaboration, responsibility, dedication, honesty, and foresight. Meanwhile, the entrepreneurial spirit can actively promote social development. For any industry, the innovation spirit is key in promoting enterprise development (Dabić et al., 2020). Superior leadership and collaboration can manage personnel effectively, strengthen cooperation, and improve enterprise development. Entrepreneurs have many obligations. For example, they should never damage public interests, and at the same time, they should maintain a unique enterprise culture and moral values. Besides, they can dedicate themselves to society through charities (Landström and Harirchi, 2018). Honesty is the foundation for enterprise development, and it should be spread through the enterprise culture, promoting the sustainable development of enterprises. With the rapid advancement of society, entrepreneurs should plan strategically with foresight, and foresight is also the full embodiment of the personal ability of entrepreneurs (Chen et al., 2016). In this article, some QS scales will be developed based on the content of entrepreneurial spirit.

### Venture Performance of New Ventures

Venture performance is the key index to measure the success or failure of a new venture. New venture performance refers to the results of new ventures after their entrepreneurial activities (Wei et al., 2020). New venture performance analysis can indicate the survival and development of the enterprises and enterprise goal accomplishment rate. This can provide a clear direction for enterprise development. Venture performance is affected by internal factors and external factors (Li et al., 2021). The external factors are complex and variable, and they refer to the environmental factors, such as market factors, policy factors, and institutional factors (Hou et al., 2017). The innovation spirit of entrepreneurs is one of the internal factors that affect venture performance. Those entrepreneurs with strong creativity are more likely to produce new ideas and can significantly improve the new venture performance. The internal factors of venture performance are mainly studied here (He and Tian, 2018; Feng and Chen, 2020).

Venture performance can be obtained through financial indices, such as sales growth rate, return on investment, and return on assets. However, since most financial indices are confidential, venture performance can also be obtained through customer satisfaction and the judgment of entrepreneurs (Goktan and Gupta, 2015). In this article, the judgment of entrepreneur can evaluate the venture performance.

### QS Scale and Data Collection of College-Student Entrepreneurs

(1) The QS scale of the college-student entrepreneur in college years

In this scale, entrepreneurial spirit is investigated for college-student entrepreneurs from five different dimensions, such as innovation spirit, leadership and cooperation, responsibility, honesty, and foresight during school. For each of these five dimensions, four questions are set, totaling 20 questions. Questions 1–4 are designed based on the actual situation of participation of students in extracurricular practice, club activities, competitions, and theoretical education, respectively, covering multiple levels of school year performance for college-student entrepreneurs. The Likert scale 5-point scoring method is chosen here: 1-point-score represents unqualified; 2-points-score represents not-so-qualified; 3-points-score represents just-qualified; 4-points-represents qualified; and 5-points-score represents well-qualified.

(2) QS scale of the college-student entrepreneur in the start-up period

The impact of innovative thinking on new ventures is analyzed based on the current situations of college-student entrepreneurs. The scale includes innovation attitude, innovation intention, and innovation behavior. Five questions are designed for each aspect. The innovation attitude can explore the attitude of entrepreneurs toward innovation during work from two aspects, namely, the spiritual attitude and material attitude. Innovation intention refers to the willingness to practice innovation during work. Innovative behavior can explore the actual innovative situation in enterprises. Likert scale 5-point scoring method is chosen here: 1-point-score represents unqualified; 2-points-score represents not-so-qualified; 3-points-score represents just-qualified; 4-points-represents qualified; and 5-points-score represents well-qualified.

### Selection of Survey Subjects and Data Analysis Methods

In this article, only college-student entrepreneurs within venture-creation time of 10 years are selected and analyzed, and data are collected both online and offline. Social software can send QS to the social media accounts of entrepreneurs online. Then, entrepreneurs can fill in the QS and feedback the results, and thus data are collected and collated. The database of the alumni association of the N College is consulted and QSs are issued to pertaining entrepreneurs for data collection. In this article, the Excel and the data analysis software SPSS22.0 are chosen for the statistical analysis of the surveyed data.

### Reliability and Validity Test Method

In this article, Q1 denotes the QS scale of college-student entrepreneurs during school years. Then, their five abilities, such as innovation spirit, leadership and cooperation ability, responsibility, honesty, and foresight are denoted from 1 to 5, respectively. Meanwhile, four questions are designed for each ability, and they are represented by 1, 2, 3, and 4, respectively. For example, the first question of innovation spirit in this scale will be expressed as Q1-1-1. Similarly, Q2 denotes the QS scale of college-student entrepreneurs during work. Then, three dimensions, such as innovation attitude, innovation intention, and innovation behavior are denoted as 1, 2, and 3, respectively. The five problems designed for each dimension are denoted as 1, 2, 3, 4, and 5, respectively. For example, the first problem of innovation attitude in the scale is expressed as Q2-1. Afterward, the reliability and validity of the Q1 and the Q2 scales will be analyzed.

The Cronbach's α coefficient can represent the reliability of the QS scale. In general, the Cronbach's α coefficient is >0.7, and the closer the coefficient gets to 1, the better the reliability of the QS scale is. Kaiser-Meyer-Olkin (KMO) and Bartlett hemispheric tests can validate the QS scales. The value of KMO should be >0.7 for the validity test.

### Research Hypothesis Analysis

Meanwhile, the innovation attitude, innovation intention, and innovation behavior are analyzed through the hypothesis test for college-student entrepreneurs. Here are the two hypotheses.

H1: Innovation attitude has a significant positive impact on innovation behavior. H2: Innovation intention has a significant positive effect on innovation behavior.

## Results and Discussion

### Reliability and Validity of the QS Scale

**Figure 2:**
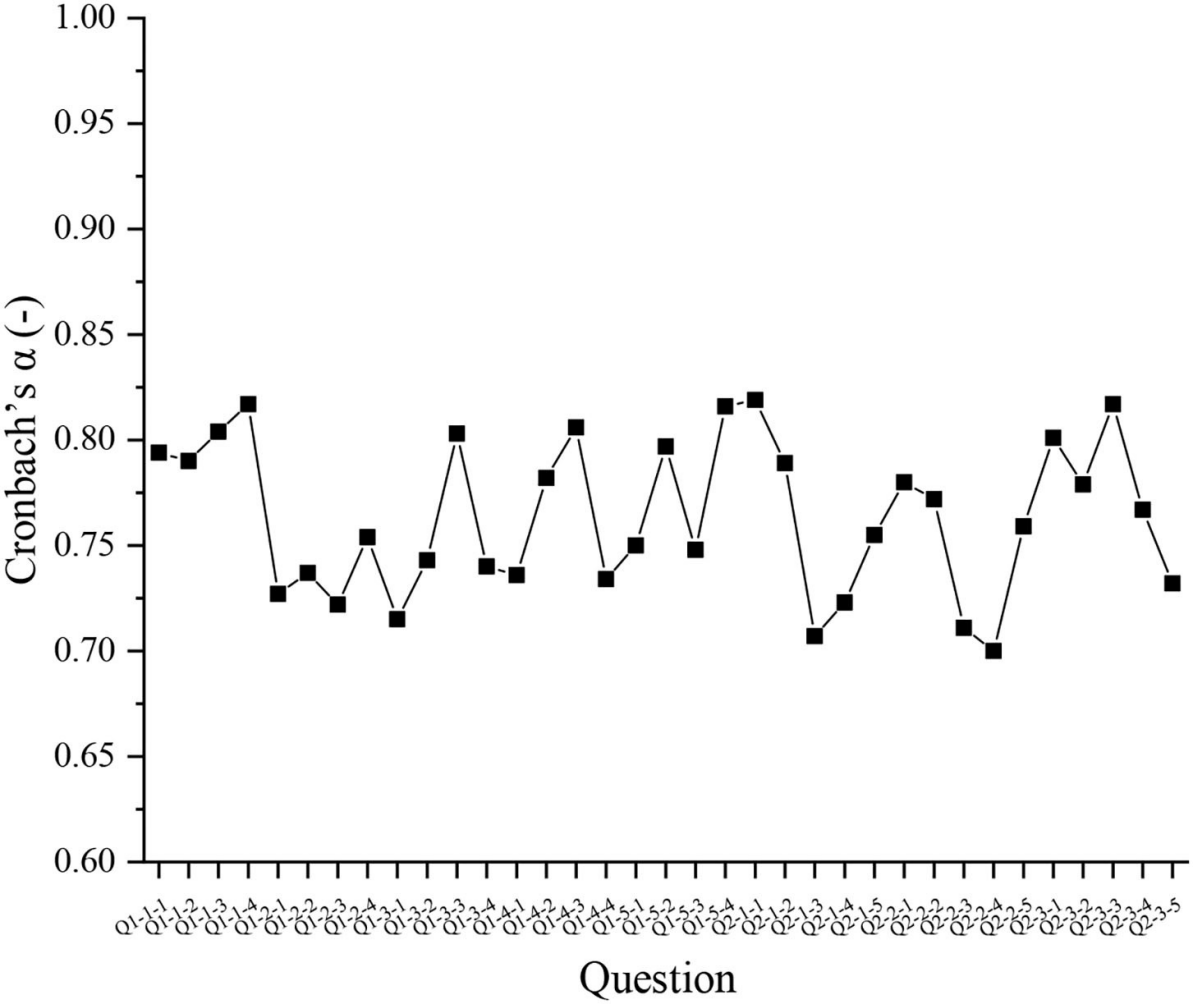
Reliability analysis of the questionnaire survey (QS) scales.

The reliability analysis results of Q1 and Q2 are shown in Figure 2.

Figure 2 indicates that the Cronbach's α coefficient of each item in the Q1 and Q2 has exceeded 0.7, indicating that the reliability of the QS scale is good and desirable.

**Figure 3:**
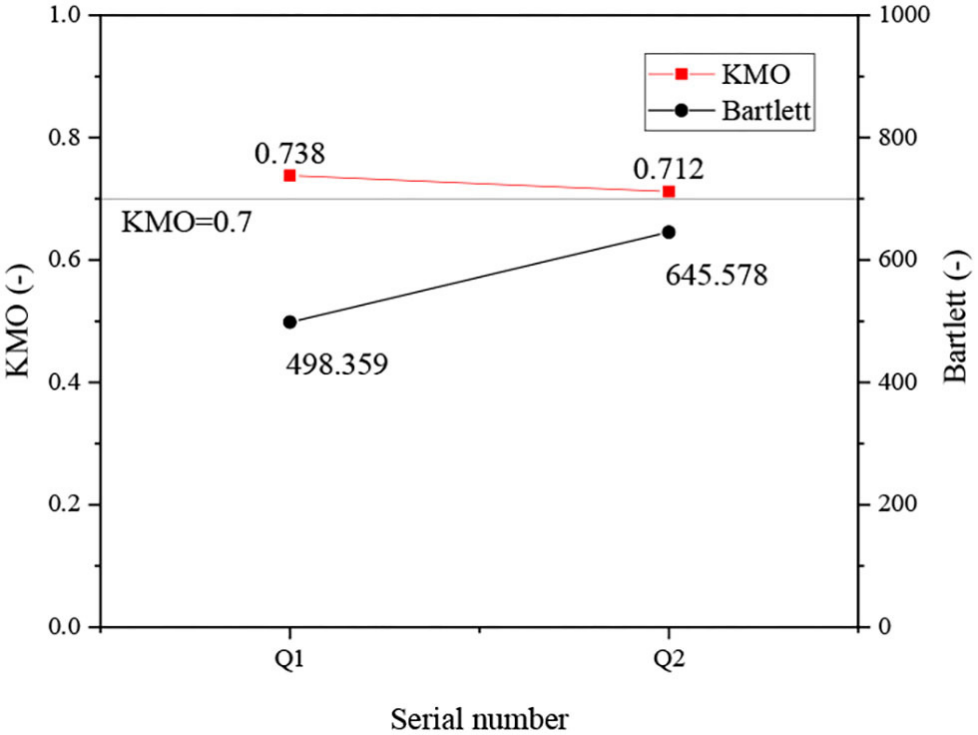
Validity analysis of the questionnaire surveys (QS) scales.

The results of the validity test are shown in Figure 3.

Figure 3 shows that the KMO values of Q1 and Q2 are 0.738 and 0.712, respectively, which are >0.07. Thus, the validity of the two QS scales is desirable.

To sum up, the designed QS scales have good reliability and validity and can be applied to practical research.

### Visualized Statistical Analysis of Surveyed College-Student Entrepreneurs

**Figure 4:**
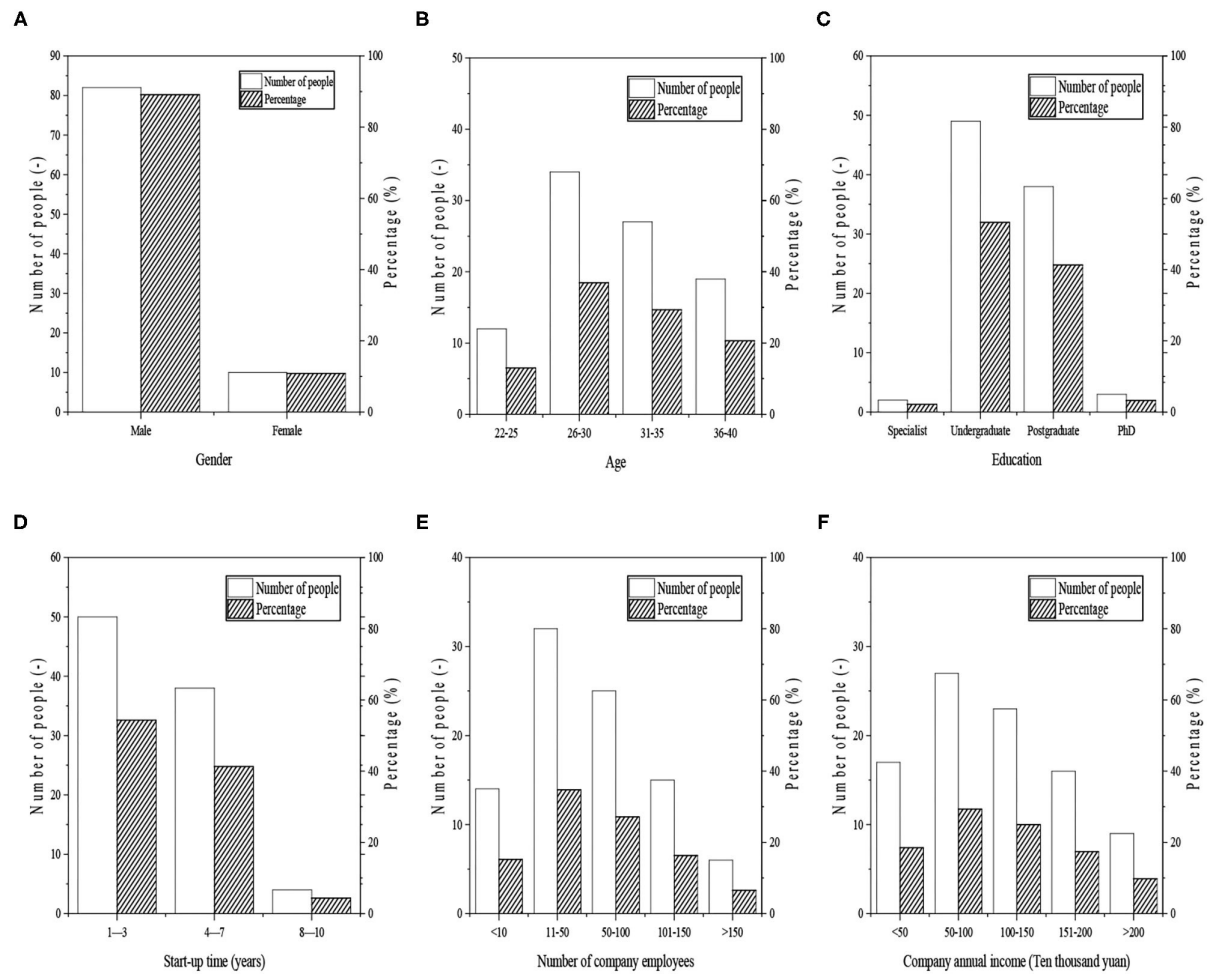
Summary and analysis of surveyed college-student entrepreneurs.

A total of 120 QSs are distributed, and 92 of them are effectively recovered, with a recovery rate of 76.7%. Six aspects are included in the QS, such as gender, age, education, venture-creation time, company size, and annual income. The specific statistical analysis results are shown in Figures 4A–F.

Figures 4A–F show analysis results of the surveyed college-student entrepreneurs from six aspects: gender, age, education, start-up time, number of company employees, and annual income of the company. The results suggest that the number of men entrepreneurs is larger than that of women entrepreneurs that include 82 males, accounting for 89.13% of the total number. While only there are 10 women, accounting for 10.87% of the total number. From the perspective of age, there are 34 entrepreneurs aged between 26 and 30, accounting for 36.96%, 27 entrepreneurs aged between 31 and 35, accounting for 29.34%, 12 entrepreneurs aged between 22 and 25, accounting for 12.03%, and 19 entrepreneurs aged between 36 and 40, accounting for 20.65%. In terms of education, most entrepreneurs have higher academic qualifications, only two of them have high school degrees, accounting for 2.17%; there are 49 entrepreneurs with a bachelor's degree, accounting for 53.26%; entrepreneurs with a master's degree reaches 38 people, accounting for 41.3%; and 3 entrepreneurs have a doctoral degree, accounting for 3.26%. From the perspective of venture-creation time, most of them are <8 years: 50 ventures have survived 1–3 years, accounting for 54.34% of the total, 38 ventures have lasted 4–7 years, accounting for 41.30%, and only four ventures have held on 8–10 years, accounting for just 4.34%. In terms of venture size, 14 ventures have <10 workers, accounting for 15.21% of the total, 32 ventures have employed 11–50 personnel, accounting for 34.78%, 25 ventures hired 50–100 employees, accounting for 27.17%, 15 companies possess 101–150 staff, accounting for 16.30%, while only six ventures have a team with 150 talents, just accounting for 16.30%. From the perspective of annual income, 17 ventures have an annual income <5,00,000, accounting for 18.48% of the total, 27 ventures have annual income between 5,00,000 and 10,00,000, accounting for 29.34%, 23 ventures have an annual income of 10,00,000–15,00,000, accounting for 25%, 16 ventures have annual income between 15,10,000 and 20,00,000, accounting for 17.39%, and only nine ventures earn more than 20,00,000 annually, just accounting for 9.78%.

Apparently, men entrepreneurs are more than women counterparts, and most entrepreneurs have received higher education and have either a bachelor's degree or higher degrees. Meanwhile, 90% of them have venture-creation time within 8 years. Most ventures are small in size, with <100 employees. Besides, over 90% of ventures have an annual income of <2 million, and most of them belong to SMEs.

### Results of the QS of College-Student Entrepreneurs During School

**Figure 5:**
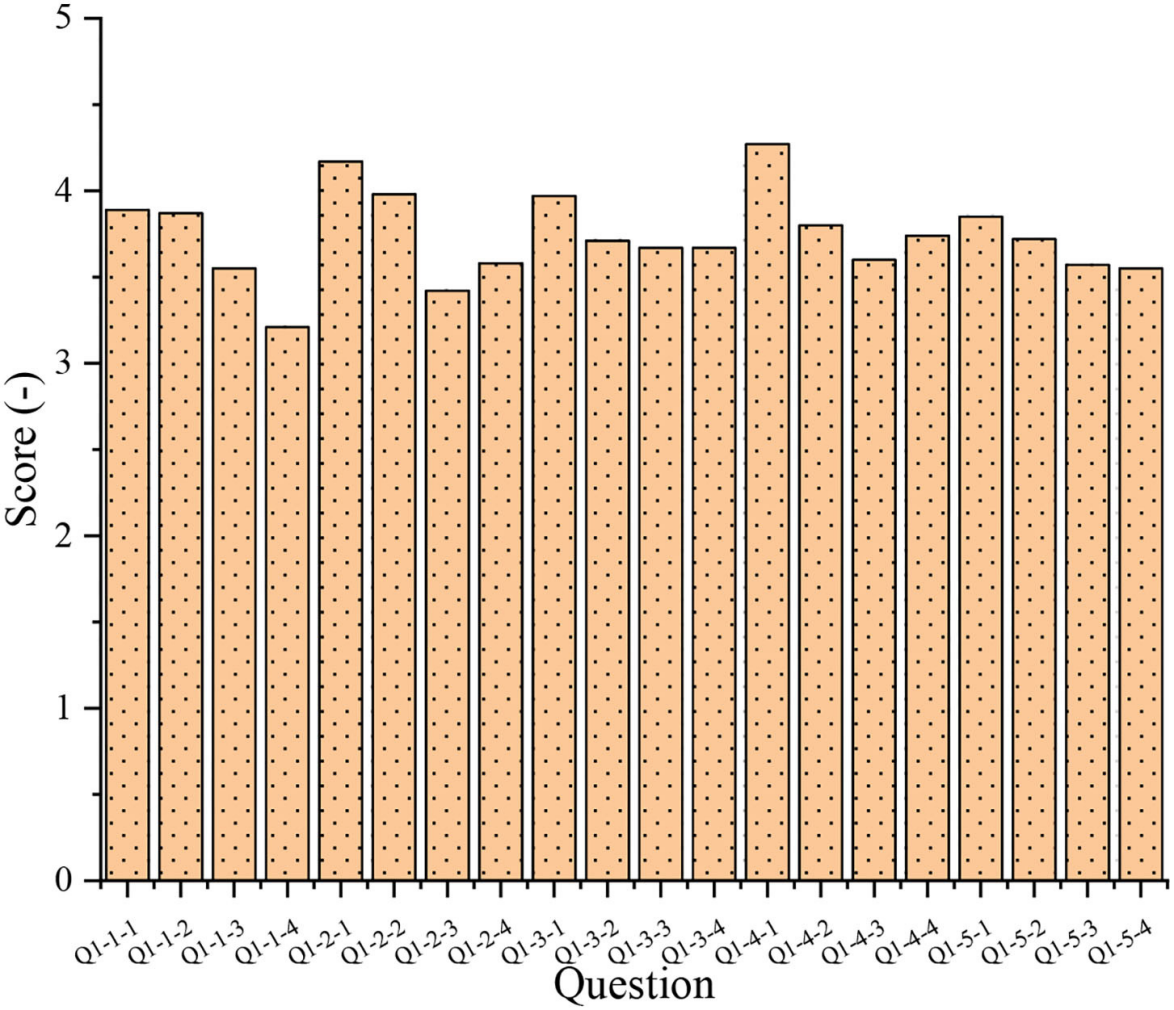
An analysis of the basic situation of college-student entrepreneurs during school.

In this article, the QS for college-student entrepreneurs is statistically analyzed, as shown in Figure 5. Figure 5 illustrate the QS results of innovation spirit, leadership and collaboration ability, responsibility, honesty, and foresight.

Accordingly, Q1-4-1 has the highest score of 4.27. Q1-1-4 had the lowest score of 3.21. In each dimension, the scores on extracurricular practice are the highest; while scores on theoretical education are the lowest. However, the innovation spirit, leadership and cooperation ability, responsibility, honesty spirit, and foresight are at a relatively high level.

Further analysis shows that during school, college-student entrepreneurs have an average score of 3.63 on innovation spirit with a variance of 0.077. Their average score of leadership collaboration ability is 3.79, and the variance is 0.090. Their average score of responsibility is 3.76, and the variance is 0.016. Their average score of honesty is 3.85, and the variance is 0.063. Their average score of foresight is 3.67, and the variance is 0.148.

**Figure 6:**
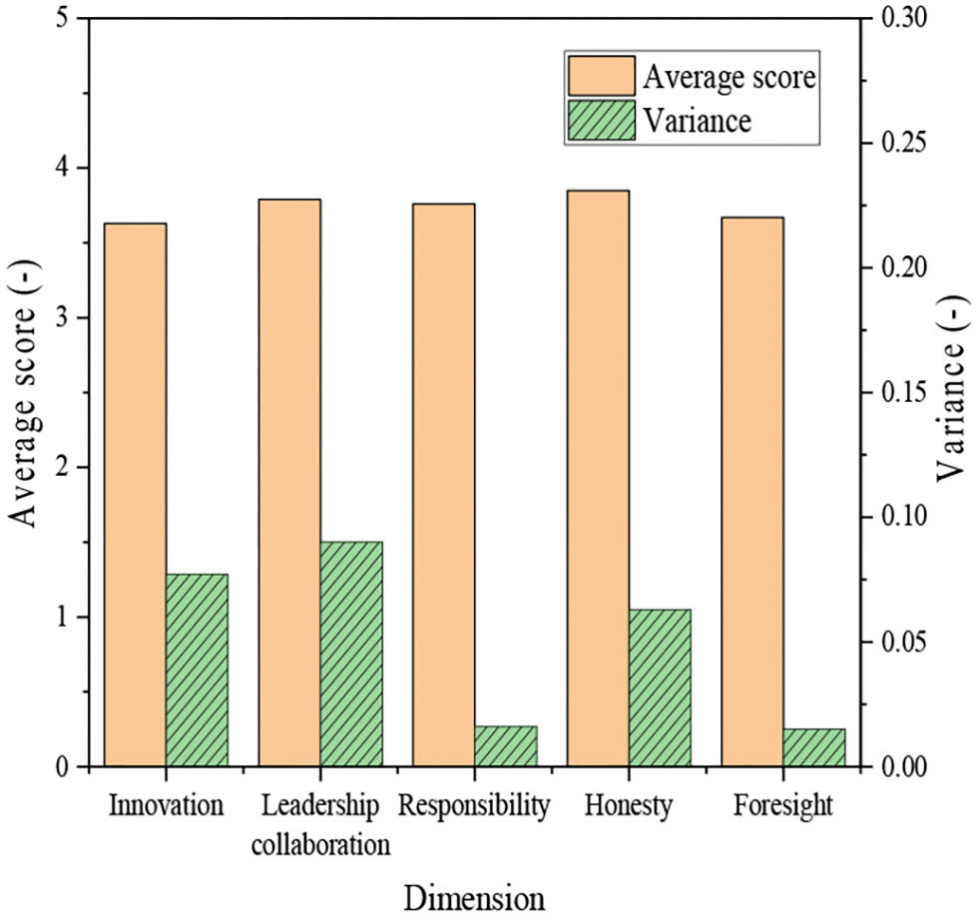
Analysis of college-student entrepreneurs during school based on five dimensions.

Further analysis of the results in Figures 5, 6 reveals that most entrepreneurs have shown high entrepreneurial spirit during their student years, which contributes greatly to the entrepreneurial activities of the entrepreneurs. Thus, the entrepreneurial spirit is very important for college students, and colleges should focus on the cultivation of entrepreneurial spirit of the students. At the same time, research shows that most entrepreneurs score higher in honesty and responsibility, proving that these two dimensions are more important in entrepreneurship and innovation education. Therefore, great importance should be attached to honesty and responsibility education.

### Results of QS on the Innovation Spirit of College-Student Entrepreneurs During Work

**Figure 7:**
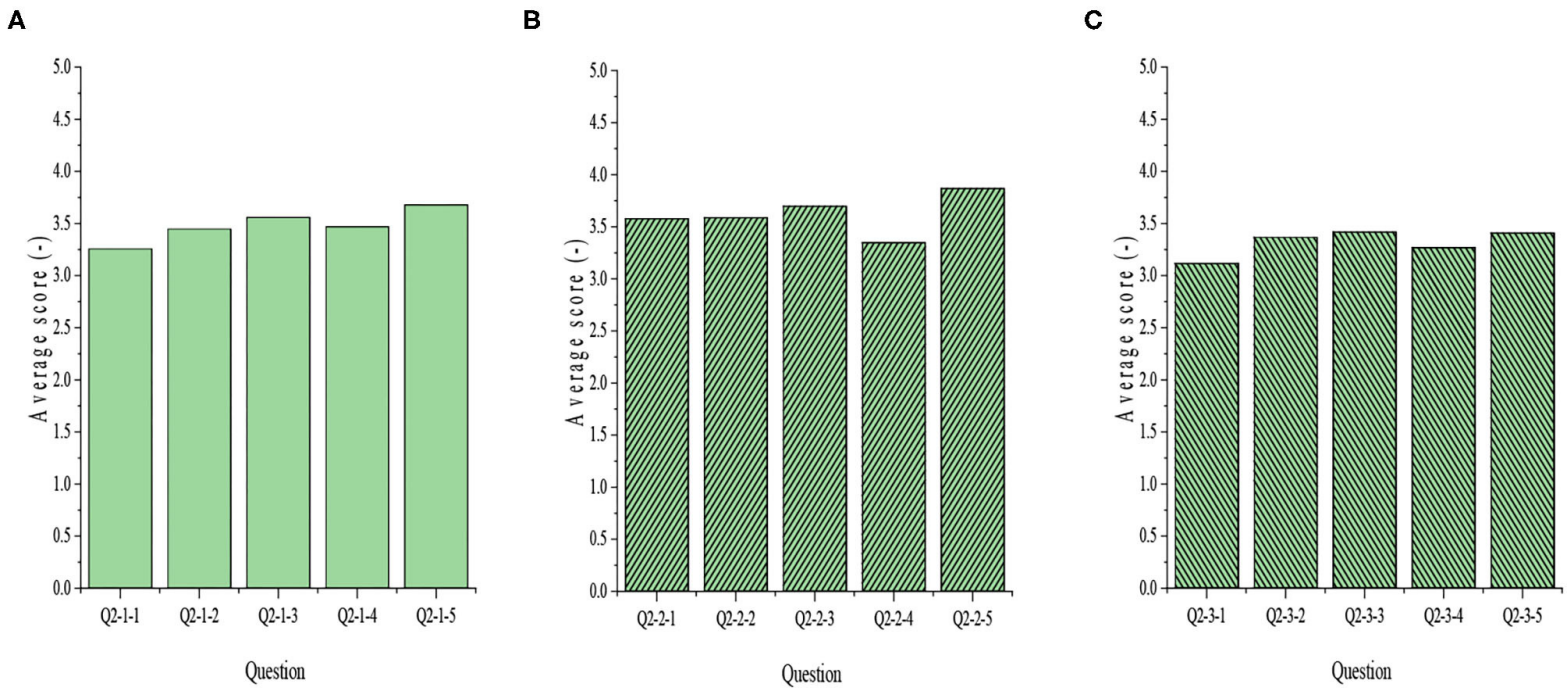
Analysis on innovation behavior of college-student entrepreneurs during work.

The results of QS on innovative attitude, innovative intention, and innovative behavior at work are shown in Figures 7A–C.

The above results show that the highest score during work is the innovation intention, which is 3.87 points; while the lowest score is the first item in the innovative behavior QS scale, with a score of 3.12.

**Figure 8:**
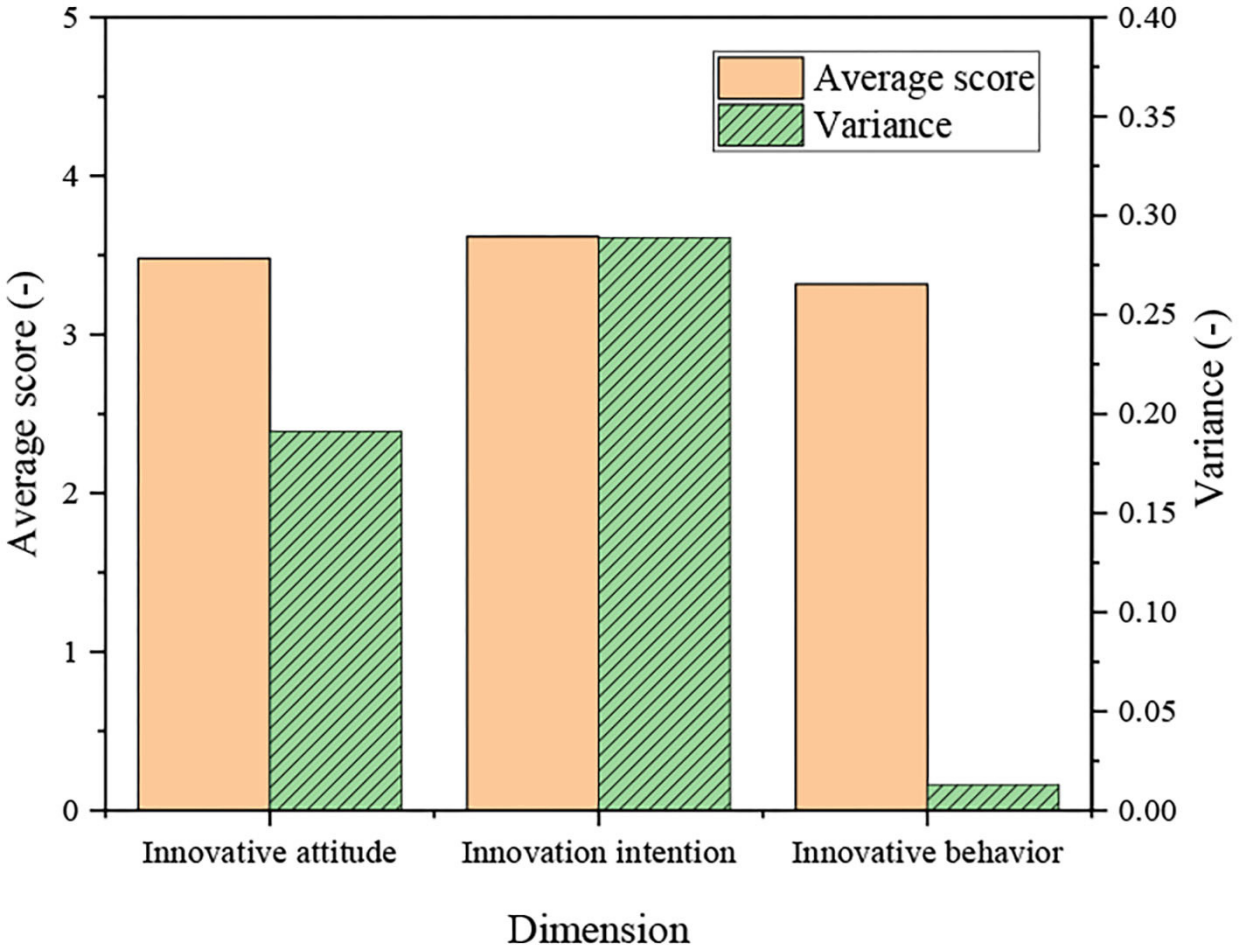
Analysis of college-student entrepreneurs during work based on three dimensions.

Figure 8 implies that the average score of innovation attitude is 3.48, and the variance is 0.019; the average score of innovation intention is 3.62, and the variance is 0.028; the average score of innovation behavior is 3.32, and the variance is 0.126.

The analysis of Figures 7A–C and Figure 8 reveal that the overall score for innovation situation of the surveyed college-student entrepreneurs is relatively high. Especially, the score of innovation intention is more than 3.5 points, and the score of innovation attitude is almost 3.5 points, indicating that they have high innovation awareness and encourage innovation in the venture development.

### Innovation Behavior of Entrepreneurs

To further study the innovation behaviors of the college-student entrepreneurs, 20 college-student entrepreneurs are randomly selected and are conducted in-depth interviews through the five dimensions QS. The in-depth interview aims to discover the innovative points, suggest innovative solutions, make reasonable arrangements, and propose innovative ways to improve the overall venture performance. After in-depth interviews, college-student entrepreneurs have chosen three cases that have been practiced most, and these results can analyze their innovation behaviors.

**Figure 9:**
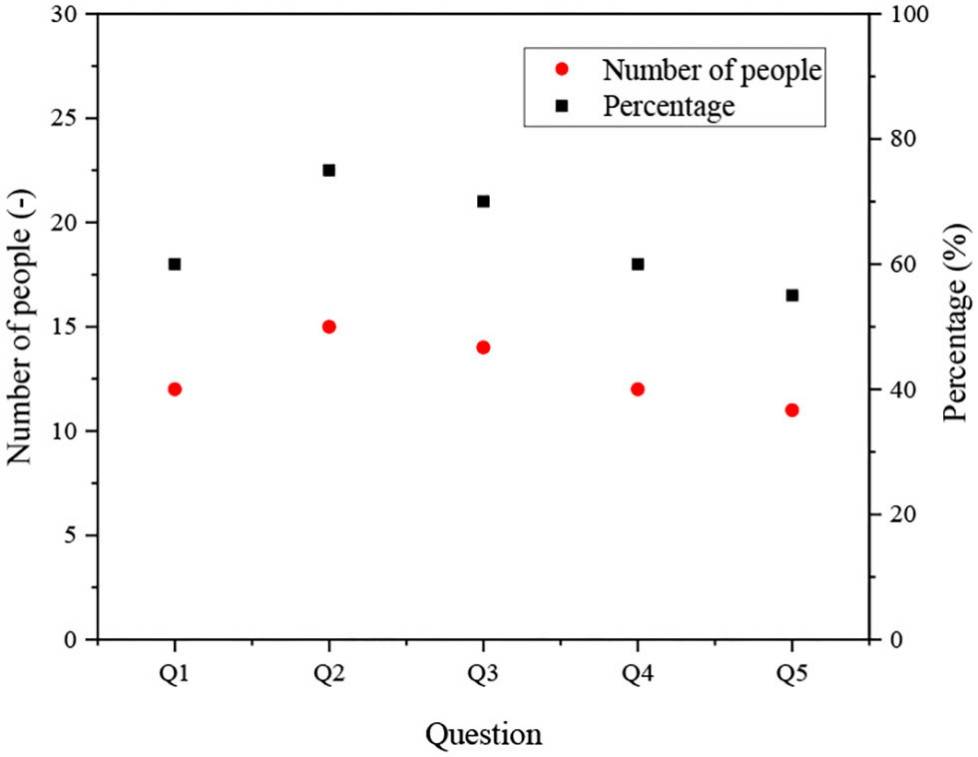
Innovation behavior analysis of the college-student entrepreneur.

Figure 9 shows that 15 college-student entrepreneurs think that they can put forward innovative solutions in their usual work. Fourteen of them think they can make reasonable arrangements for the implementation of ideas. At the same time, 13 of them believe that they have put forward innovative methods in their work that can improve job performance.

Further analysis implies that the results of in-depth interviews reflect the importance of innovation spirit in venture development. Innovation spirit can effectively improve work efficiency and venture performance, promote venture development, and bring vitality to ventures.

In this article, the SPSS22.0 is chosen to analyze the results of the data *t*-test. A significant *t*-test is conducted for these two hypotheses, as shown in Table 1.

**Table 1:** Hypothesis test results.

**Number**	***t***	**Test result**
H1	8.674	Established
H2	7.147	Established

Accordingly, the hypotheses of H1 and H2 are valid. The higher the innovation attitude and intention of college-student entrepreneur are, the stronger the innovation behavior is.

## Conclusion

In this article, the QS method is utilized to investigate and analyze the basic situation of young entrepreneurs during their school and start-up periods, and the following conclusions are obtained: according to the QS results, there are more men than women in college-student entrepreneurs who have received higher education, and most ventures are very young and developing rapidly. In the QS scales, the highest score appeared in honesty, the score of extracurricular practice is the highest. Most entrepreneurs show remarkable entrepreneurial spirit when they are students. Therefore, schools should focus on the cultivation of innovation spirit and honesty in extracurricular practice activities and community activities. At work, most enterprises have obvious innovation attitudes and innovation intentions. The in-depth interviews of 20 entrepreneurs show that most entrepreneurs put forward innovative solutions in their daily work and think that putting forward innovative behavior can effectively improve the work performance and venture performance, while innovation attitude and innovation intention have a positive impact on the innovation behavior. The survey data are rich, and the entrepreneurial spirit of college-student entrepreneurs is analyzed from different angles. The results can provide an effective reference for college entrepreneurship and innovation education and contribute to the research on entrepreneurship and innovative behavior. However, due to time constraints, most of the respondents have been chosen from the same college. The survey sample lacks universality, which affects the application value of the research results. In the follow-up study, the scope of the study should be expanded, and the entrepreneurs of different colleges should be contained for more reliable research results.

## Data Availability Statement

The raw data supporting the conclusions of this article will be made available by the authors, without undue reservation.

## Ethics Statement

The studies involving human participants were reviewed and approved by Ningbo University Ethics Committee. The patients/participants provided their written informed consent to participate in this study. Written informed consent was obtained from the individual(s) for the publication of any potentially identifiable images or data included in this article.

## Author Contributions

All authors listed have made a substantial, direct and intellectual contribution to the work, and approved it for publication.

## Conflict of Interest

The authors declare that the research was conducted in the absence of any commercial or financial relationships that could be construed as a potential conflict of interest.

## Publisher's Note

All claims expressed in this article are solely those of the authors and do not necessarily represent those of their affiliated organizations, or those of the publisher, the editors and the reviewers. Any product that may be evaluated in this article, or claim that may be made by its manufacturer, is not guaranteed or endorsed by the publisher.
